# Identification of novel aspartic proteases from *Strongyloides ratti *and characterisation of their evolutionary relationships, stage-specific expression and molecular structure

**DOI:** 10.1186/1471-2164-10-611

**Published:** 2009-12-16

**Authors:** Luciane V Mello, Helen O'Meara, Daniel J Rigden, Steve Paterson

**Affiliations:** 1 School of Biological Sciences, University of Liverpool, Crown Street, Liverpool L69 7ZB, UK; 2 Department of Pharmacology and Therapeutics, University of Liverpool, Ashton Street, Liverpool, L69 3GE, UK

## Abstract

**Background:**

Aspartic proteases are known to play an important role in the biology of nematode parasitism. This role is best characterised in blood-feeding nematodes, where they digest haemoglobin, but they are also likely to play important roles in the biology of nematode parasites that do not feed on blood. In the present work, we investigate the evolution and expression of aspartic proteases in *Strongyloides ratti*, which permits a unique comparison between parasitic and free-living adult forms within its life-cycle.

**Results:**

We identified eight transcribed aspartic protease sequences and a further two genomic sequences and compared these to homologues in *Caenorhabditis elegans *and other nematode species. Phylogenetic analysis demonstrated a complex pattern of gene evolution, such that some *S. ratti *sequences had a one-to-one correspondence with orthologues of *C. elegans *but that lineage-specific expansions have occurred for other aspartic proteases in these two nematodes. These gene duplication events may have contributed to the adaptation of the two species to their different lifestyles. Among the set of *S. ratti *aspartic proteases were two closely-related isoforms that showed differential expression during different life stages: ASP-2A is highly expressed in parasitic females while ASP-2B is predominantly found in free-living adults. Molecular modelling of the ASP-2 isoforms reveals that their substrate specificities are likely to be very similar, but that ASP-2B is more electrostatically negative over its entire molecular surface than ASP-2A. This characteristic may be related to different pH values of the environments in which these two isoforms operate.

**Conclusions:**

We have demonstrated that *S. ratti *provides a powerful model to explore the genetic adaptations associated with parasitic versus free-living life-styles. We have discovered gene duplication of aspartic protease genes in *Strongyloides *and identified a pair of paralogues differentially expressed in either the parasitic or the free-living phase of the nematode life-cycle, consistent with an adaptive role for aspartic proteases in the evolution of nematode parasitism.

## Background

Adaptation of nematode taxa to a parasitic life-style represents an evolutionary challenge that is likely to be met, in part, by gene duplication in a gene family and subsequent acquisition of novel gene function among its paralogous members. One group of genes that has received considerable interest in this respect are aspartic proteases. Aspartic proteases are defined by having catalytic aspartic acid residues located in their active site clefts and include pepsins, renins, cathepsins D and E and chymosins [1-3]. In parasitic nematodes, aspartic proteases have been associated with digestion of host haemoglobin in the trichostrongylid *Haemonchus contortus *and in the hookworms *Ancylostoma caninum *and *Necator americanus *[4]. Moreover, the aspartic proteases of *A. caninum *and *N. americanus *each exhibit specificity in their ability to digest haemoglobin from natural versus unnatural hosts, indicating evolutionary adaptation to their host species [4]. Consequently, several aspartic proteases are currently being developed as vaccines in trichostrongylids and hookworms, many of which demonstrate an ability to reduce worm burden and/or egg output [5,6]. Aspartic proteases have also been shown to degrade skin macromolecules and aid skin penetration in hookworms, suggesting that their role in nematode parasitism is not limited to digestion of haemoglobin [7]. Given this, the aspartic proteases may play a role in the biology of nematode parasites that do not feed on blood, such as *Strongyloides stercoralis*, *Onchocerca volvulus *and *Brugia malayi *in which aspartic proteases have previously been identified [7,8].

**Figure 1:**
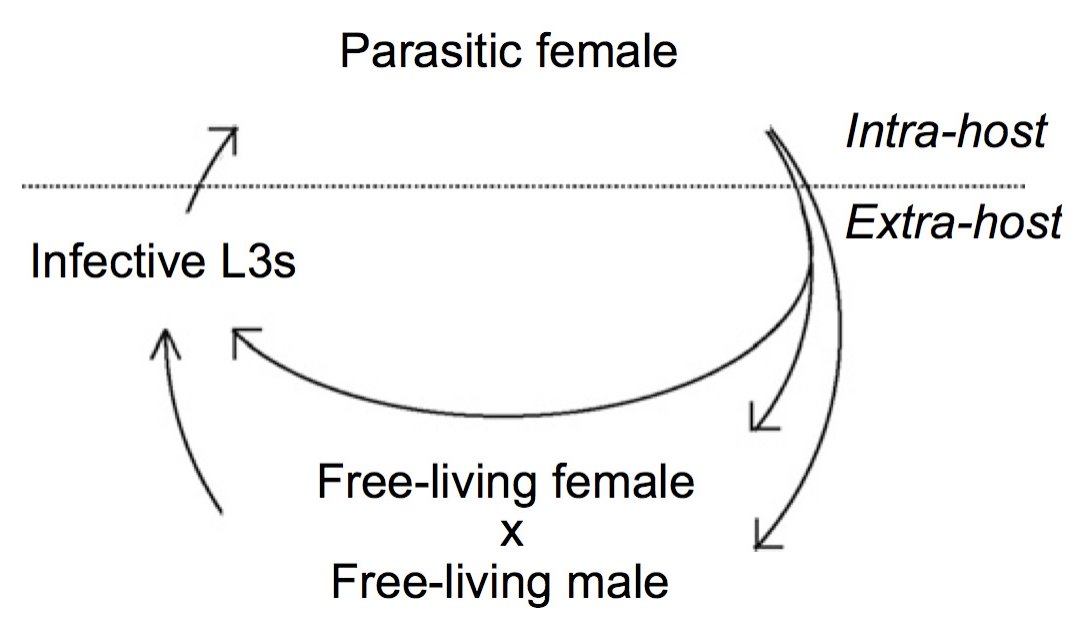
**Life-cycle of *S. ratti***. Eggs produced by mitotic parthenogenesis by parasitic females can develop either directly into iL3s by homogonic development or into free-living males and females, which reproduce sexually to produce eggs that develop into iL3s.

Nematodes of the genus *Strongyloides *infect a wide range of mammalian species, including humans and livestock, and infection occurs by skin penetration. *Strongyloides *spp. is not blood-feeding and parasitism is believed to have arisen separately in the lineages leading to *Strongyloides *and to the hookworms and trichostrongylids [9]. *Strongyloides *provides a useful model to study the evolution of genes associated with parasitism, since both parasitic and free-living adults are present in the life-cycle (Figure 1). Thus, one might predict that genes, including aspartic proteases, which retain distinct functions in free-living and parasitic environments, are maintained within the *Strongyloides *genome. Previously, and in support of this prediction, we have presented preliminary evidence that distinct aspartic proteases from *S. ratti*, a laboratory model of *Strongyloides *infection, were differentially transcribed between free-living females and parasitic females [10].

In the present study we have identified ten aspartic proteases in *S. ratti *using sequence data from a collection of 14,761 cDNA clones described in Thompson *et al*. [11] and Evans *et al*. [10], by novel pyrosequencing of normalised cDNA using a 454-FLX sequencer and from an early draft genome sequence of *S. ratti *http://www.sanger.ac.uk/sequencing/Strongyloides/. We used comparative sequence analysis and phylogenetic reconstruction between these and other aspartic proteases, including those belonging to the non-parasitic nematodes *Caenorhabditis elegans *and *C. briggsae*, to identify separate duplication events within different nematode lineages. We identified a pair of paralogous aspartic proteases in *S. ratti *(which we term ASP-2A and ASP-2B) that have arisen as a result of a recent duplication event but for which ASP-2A is highly expressed in parasitic females while ASP-2B is highly expressed in free-living females, and we present a molecular model that allows this pair of paralogous genes to be placed in a structural context.

## Results

### Identification of aspartic protease sequences in *S. ratti*

**Figure 2:**
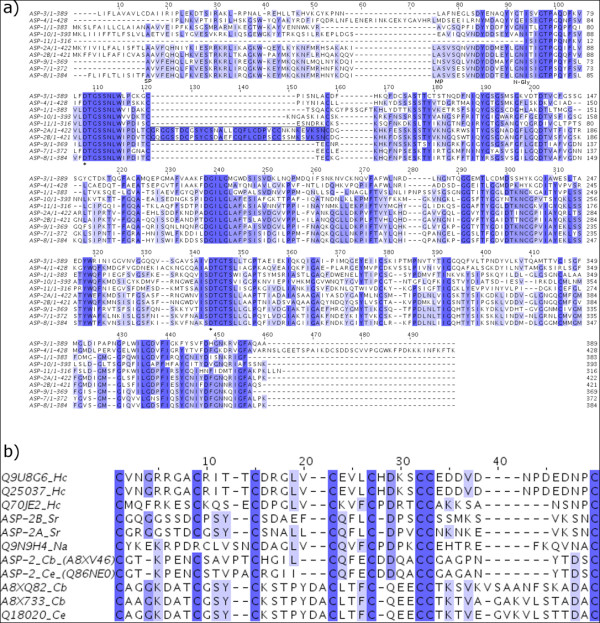
**Protein sequence alignment of aspartic proteases**. (a) Derived protein sequences of the 10 S. ratti aspartic protease aligned using MUSCLE [38]. Predicted signal peptide, the mature protein starting point, and conserved N-glycosylation sites are labelled SP, MP and N-Gly, respectively. The two catalytic aspartic acid residues are indicated by (*). Cys-rich region indicated by a box. (b) Alignment of the Cys-rich region from all species using MUSCLE [38]. Organisms are indicated after the accession numbers, as followed: Cb, *Caenorhabditis briggsae*; Ce, *Caenorhabditis elegans*; Hc, *Haemonchus contortus*; Na; *Necator americanus*; Sr, *Strongyloides ratti*. Figures were generated using JALVIEW [39].

BLASTx searches of 3,688 contigs from *S. ratti *EST sequences (cDNA library) and 29,137 contigs from the pyrosequencing against the Wormpep, UniprotKB/Swiss-Prot and nr databases revealed hits against various aspartic protease proteins. Upon close inspection of the consensus sequence for each contig, eight transcripts of aspartic protease were identified, which we termed ASP-1, ASP-2A, ASP-2B, ASP-3, ASP-4, ASP-7, ASP-9, and ASP-10. Two further putative asparatic protease genes, *asp-8 *and *asp-11*, were identified within the draft genome sequence for *S. ratti *(i.e. predicted genes for which there was no support from transcript evidence). 5' RACE was used to complete full length coding sequences for ASP-2A and ASP-2B, while the full length for other transcripts were obtained using the genome sequences for *S. ratti*, when necessary. The only exceptions were ASP-3 and ASP-4 where the present state of the genome sequence did not allow for completion at the N-terminus. Both nucleotide and amino-acid differences were observed across the length of all 10 sequences, indicating these isoforms represented distinct, paralogous loci rather than splice-site variants. Orthology was determined by the phylogenetic analysis and nomenclature was assigned to each gene, where possible, based on its orthology with the published *C. elegans *aspartic proteases, ASP-1 to ASP-6 [12] and to uncharacterised *C. elegans *aspartic proteases. Due to the difficulty in assigning orthologous *S. ratti *transcripts to *C. elegans *ASP-5 and ASP-6 based on our phylogenetic analysis (Cluster III discussion below), numbers 5 and 6 were not used. The eight *S. ratti *aspartic protease genes with their predicted amino acid sequences were submitted to GenBank (GenBank:FJ756439, GenBank:FJ756440, GenBank:FJ756441, GenBank:FJ756442, GenBank:FJ756443, GenBank:FJ756444, GenBank:FJ756445, and GenBank:FJ756446). All 454 cDNA sequence data are available from *NEBC Envgen catalogue *http://nebc.nox.ac.uk/data/envbase. A Muscle alignment of the predicted amino acid sequences for each *S. ratti *aspartic protease is shown in Figure 2a. Signal peptides of the pre-proenzymes were determined and labelled in the alignment. It is known that aspartic proteases undergo a post-translational modification whereby about 60 residues are cleaved from the N-terminus of the proenzyme producing the mature protein and the predicted cleavage points of these proteins are also marked in the alignment (Figure 2a). N-glycosylation site, presupposed to be necessary for lysosomal targeting, are also labelled, and as for *C. elegans*, the site is absent in ASP-3 and ASP-4. Syntichaki *et al*. [13] have shown these two aspartic proteases are involved in neurodegeneration in *C. elegans*.

### Phylogenetic analysis of aspartic proteases

**Figure 3:**
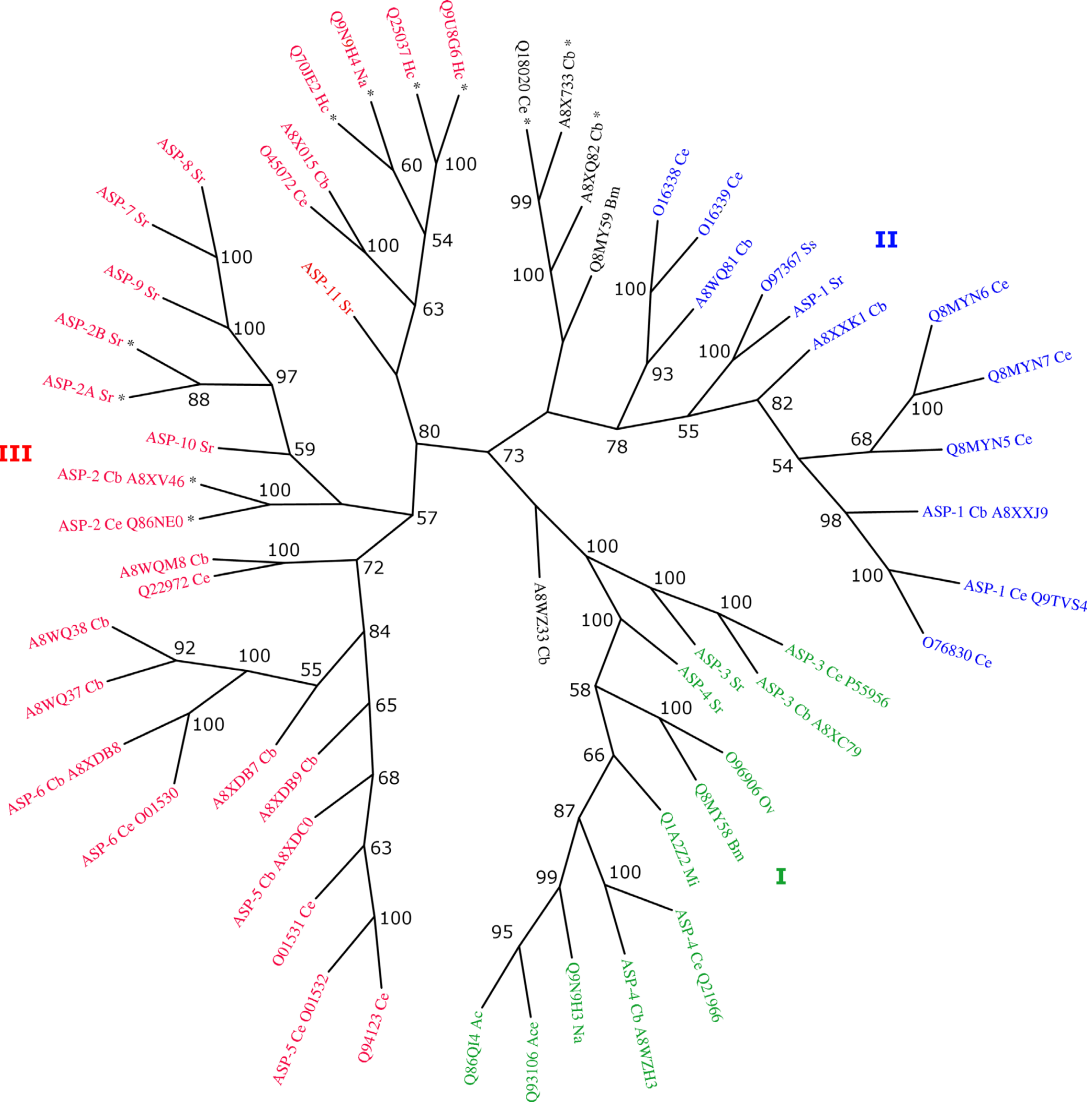
**Unrooted phylogenetic tree**. The eukaryotic aspartic protease domain, PF00026 was searched against the PFAM database [37] and sequences under the nematode branch were retrieved. Sequences were aligned using MUSCLE [38] and highly similar sequences were removed from the alignment. An unrooted neighbor-joining tree was calculated from protein distances using the MEGA package [40,41]. Gapped regions were excluded in a complete fashion and percentage bootstrap values from 500 replicates were derived. Numbers above branches indicate the relative frequency in which bipartitions were observed (only values above 50% are shown). Sequences used in the final alignment are labelled according to their accession number at UNIPROT. Organisms are indicated after the accession numbers, as followed: Ac, *Ancylostoma caninum*; Ace, *Ancylostoma ceylanicum*; Bm, *Brugia malayi*; Cb, *Caenorhabditis briggsae; *Ce, *Caenorhabditis elegans; *Hc, *Haemonchus contortus*; Mi, *Meloidogyne incognita*; Na; *Necator americanus*; Ov, *Onchocerca volvulus*; Sr, *Strongyloides ratti*; Ss, *Strongyloides stercoralis*. Major groups I-III referred to in the text and coloured differently. All the sequences which present the Cys-rich insertion are labelled with a star.

Protein distance-based phylogenetic analysis of the eukaryotic aspartic protease amino acid sequences resulted in an unrooted tree with three clusters (Figure 3). The *C. elegans *sequences for aspartic proteases were used as reference for the cluster division and interpretation as follows; Cluster I contained both ASP-3 and ASP-4; Cluster II *C. elegans *ASP-1; and Cluster III contained ASP-2, ASP-5 and ASP-6 sequences. The tree could also be interpreted as two major groups, one containing ASP-1, ASP-3, ASP-4, and the other ASP-2, ASP-5 and ASP-6. Two sequences did not group with any other; Q8 MY59 from *B. malayi *and A8WZ33, an uncharacterised transcript in *C. briggsae*. There are also three uncharacterised transcript sequences, two from *C. briggsae *(A8X733 and A8XQ82) and one from *C. elegans *(Q18020) which forms a small group. Interestingly, the tree topology can be correlated with genomic position: in *C. elegans*, the ASP-1, ASP-2, ASP-5 and ASP-6 are located on chromosome V, whilst ASP-3 and ASP-4 are on chromosome X [12]. In the cases of branches containing *C. elegans *ASP-3 and ASP-4, each species is represented by a single sequence. This clear evolutionary relationship allows for the naming of the corresponding *S. ratti *transcripts.

A similar pattern was observed for ASP-1, although this gene is not well represented in terms of nematode species. As expected, ASP-1 from *S. ratii *and *S. stercoralis *are very similar, as shown by the tree. Remarkably, in the group containing *C. elegans *ASP-1 and four other hypothetical *C. elegans *proteins, *C. briggsae *is represented by ASP-1 alone. This suggests that there have been a number of duplications in the ASP-1 lineage in *C. elegans *since its divergence from *C. briggsae*. This scenario is supported by the fact that three of the *C. elegans *hypothetical proteins are genomic neighbours (Q8 MYN5, Q8 MYN6 and Q8 MYN7).

A different scenario was observed for the ASP-2, ASP-5 and ASP-6 *C. elegans *sequences, the latter two of which are contained within a monophyletic group composed exclusively of *Caenorhabditis *sequences. This branches with a group of six *S. ratti *sequences, also clearly sharing a recent common origin. In a further branch are located two *H. contortus *sequences [14] which also group together. This suggests that at least three independent duplications have occurred in this part of the protease family, one in the lineage containing *C. elegans*, another in the predecessor of *H. contortus*, and a distinct set of events in the branch leading to *S. ratti*. Similar results were also obtained using minimum evolution and maximum parsimony phylogenetic approaches (not shown). These results mean that the naming of *S. ratti *sequences by orthology with their *C. elegans *counterparts breaks down here. Note that for other species other than *C. elegans*, numbering of aspartic proteases does not appear to be related to evolutionary history, instead being based, for example, on the order in which genes were discovered.

### Analysis of the ASP-2A and ASP-2B protein sequences

A pair of paralogous *S. ratti *sequences, which we termed ASP-2A and ASP-2B, exhibited 76% amino-acid similarity to each other and shared a conspicuous characteristic with *C. elegans *ASP-2: the presence of a cysteine rich insertion (see Figure 2a) near the beginning of the sequence, which consists of ~30-35 amino acids located between the two putative catalytic residues [15]. Although these *S. ratti *sequences do not form a monophyletic cluster with *C. elegans *ASP-2, we named them ASP-2A and ASP-2B in recognition of this shared cysteine rich insertion. Of those genes represented in our phylogenetic analysis, the Cys-rich insertion was present in: Q88NE0 and Q18020 of *C. elegans*; A8XV46, A8X733 and A8XQ82 of *C. briggsae*; Q9N9H4 of *N. americanus*; Q70JE2, Q25037 and Q9U8G6 of *H. contortus; *and ASP-2A and ASP-2B of *S. ratti*. Interestingly, the Cys-rich insertion has a quite sporadic distribution: Q18020, A8X733 and A8XQ82 form the small isolated group mentioned above, while all the other sequences grouped within Cluster III of the phylogeny presented in Figure 3. Alignment of these sequences indicated that all Cys-rich insertions from these proteins shared a common ancestor: all contained 6 cysteine residues. With the exception of orthologous pairs from *C. elegans *and *C. briggsae *(Q86NE0 and A8XV46), a very recently duplicated pair from *C. briggsae *(A8XQ82 and A8X733) and the two *H. contortus *sequences (Q25037 and Q9U8G6), sequence identity between insertions was low at 21-39% (Figure 2b). An alignment also indicated that Q88NE1 in *C. elegans *was identical to Q88NE0 except for the addition of ~200 residues at the N-terminal (result not shown). However, it is unlikely that this longer protein is actually expressed. First, protein domain analysis reveal the presence of a partial eukaryotic aspartic protease domain coded for by the extra N-terminal residues, but structural analysis suggests that the N-terminal half-domain would be unlikely to fold stably, since it lacks key components of the protein core. Also, a BLASTn search against the collection of *C. elegans *ESTs failed to reveal a single sequence spanning the junction between the additional N-terminal region and the complete aspartic protease domain common to Q88NE1 and Q86NE0. For *H. contortus*, Q9U8G6 and Q25037 share 98% sequence identity and so are not likely to be true isoforms, but instead duplicated entries within UniProt. By contrast, *H. contortus *Q25037 and Q70JE2 share 50% amino-acid sequence identity, and so are likely to represent distinct, paralogous loci.

### Analysis of differential transcription of aspartic proteases by rtPCR analysis

**Figure 4:**
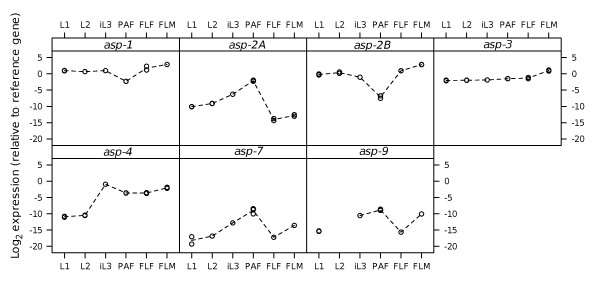
**Transcription levels of the *S. ratti *aspartic proteases**. The change in transcription levels of asp-1, asp-2A, asp-2B, asp-3, asp-4, asp-7 and asp-9 through different developmental stages measured by real-time PCR. Transcription levels for each stage are given as a log_2 _ratio relative to the reference gene (rpl20). The values of each biological replicate are plotted as a mean of of 3 technical replicates.

Transcription of seven aspartic protease genes across the life cycle of *S. ratti *was measured by rtPCR (ASP-10 from the pyrosequencing analysis and aspartic proteases inferred by homology with the *S. ratti *genome, ASP-8 and ASP-11, were excluded). Figure 4 shows the relative expression of each gene between different life developmental stages. While transcription was observed for every gene in all stages, the expression of each transcript was variable between developmental stages. ASP-1 and ASP-3 were found to be the most constitutively transcribed in *S. ratti*. In *C. elegans*, ASP-1 expression is lower in adults than in larvae whereas *C. elegans *ASP-3 expression is relatively constitutive through development http://www.wormbase.org. *S. ratti *ASP-4 transcription was markedly lower in the immature L1 and L2 stages than in iL3s or in any of the three adult stages, which is a similar pattern to that seen for *C. elegans *ASP-4 [16]. *S. ratti *ASP-7 and ASP-9 exhibited lower overall abundance than for the other *S. ratti *aspartic protease transcripts, and with levels somewhat elevated in the parasitic females (PAF) and the free living males (FLM). Pronounced differences in transcription levels between developmental stages were observed for the two *S. ratti *ASP-2 isoforms; with ASP-2A levels highest in the parasitic females (PAF) and lowest in the free living adults (FLF and FLM), whereas ASP-2B levels were lowest in PAF when compared with FLF and FLM. By contrast, *C. elegans *ASP-2 exhibits increasing expression from embryo through to adult [16].

### Molecular Modelling

**Figure 5:**
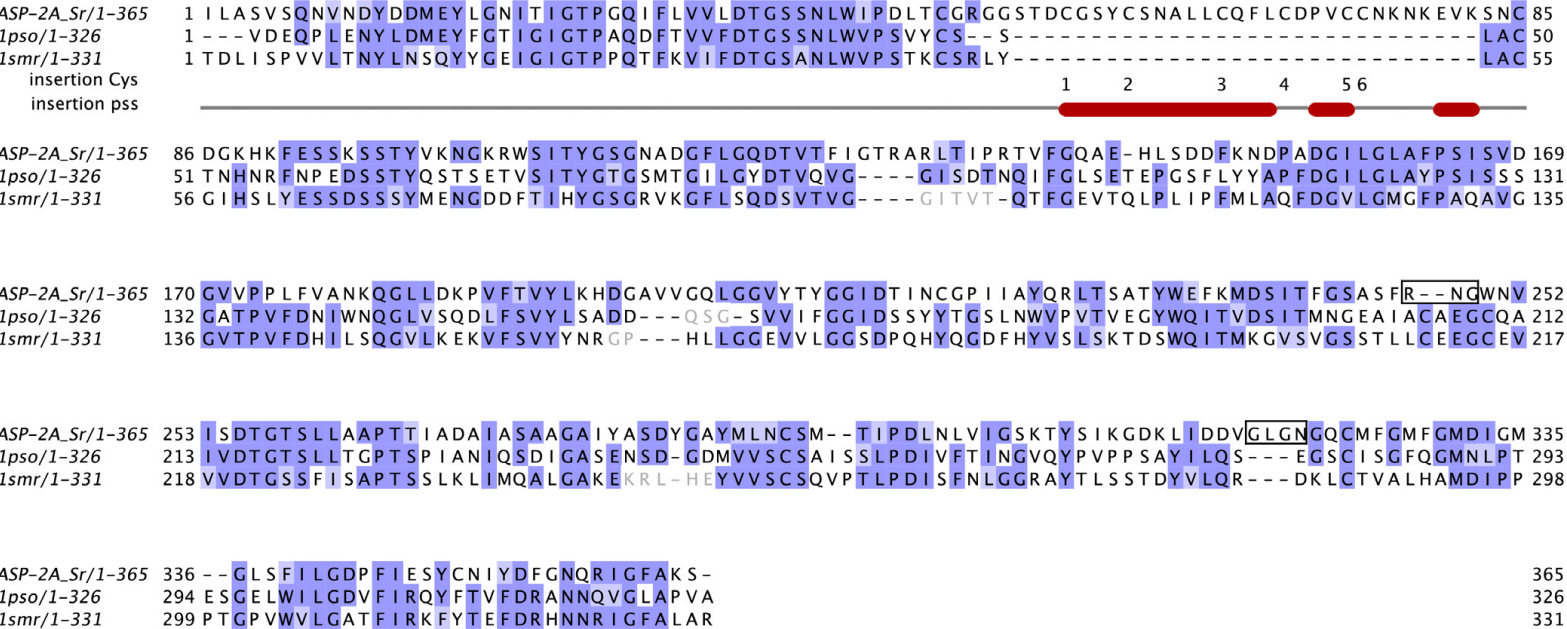
**Protein sequence alignment between *S. ratti *ASP-2A and the principal templates used in its modelling**. Alignment of ASP-2A with the principal templates used in its modelling, human pepsin in complex with pepstatin (PDB code 1pso; [44]) and mouse renin bound to the decapeptide inhibitor CH-66 (1smr; [45]). Shading indicates the degree of sequence similarity at each position. For the Cys-rich region, Cys residues are numbered and secondary structure predicted (using PSIPRED [53]; red indicates α-helices) on lines below the alignment. Where only a single one of these two templates was used, the excluded template region is shown in light grey text. Boxed regions of ASP-2A indicate portions modelled using additional templates, *Candida albicans *aspartic proteinase 3 (PDB code 2h6t; [46]) for residues 247-249 and human renin (PDB code 1hrn; [47]) for residues 308-311 (see Materials and Methods). The figure was produced with Jalview [39].

**Figure 6:**
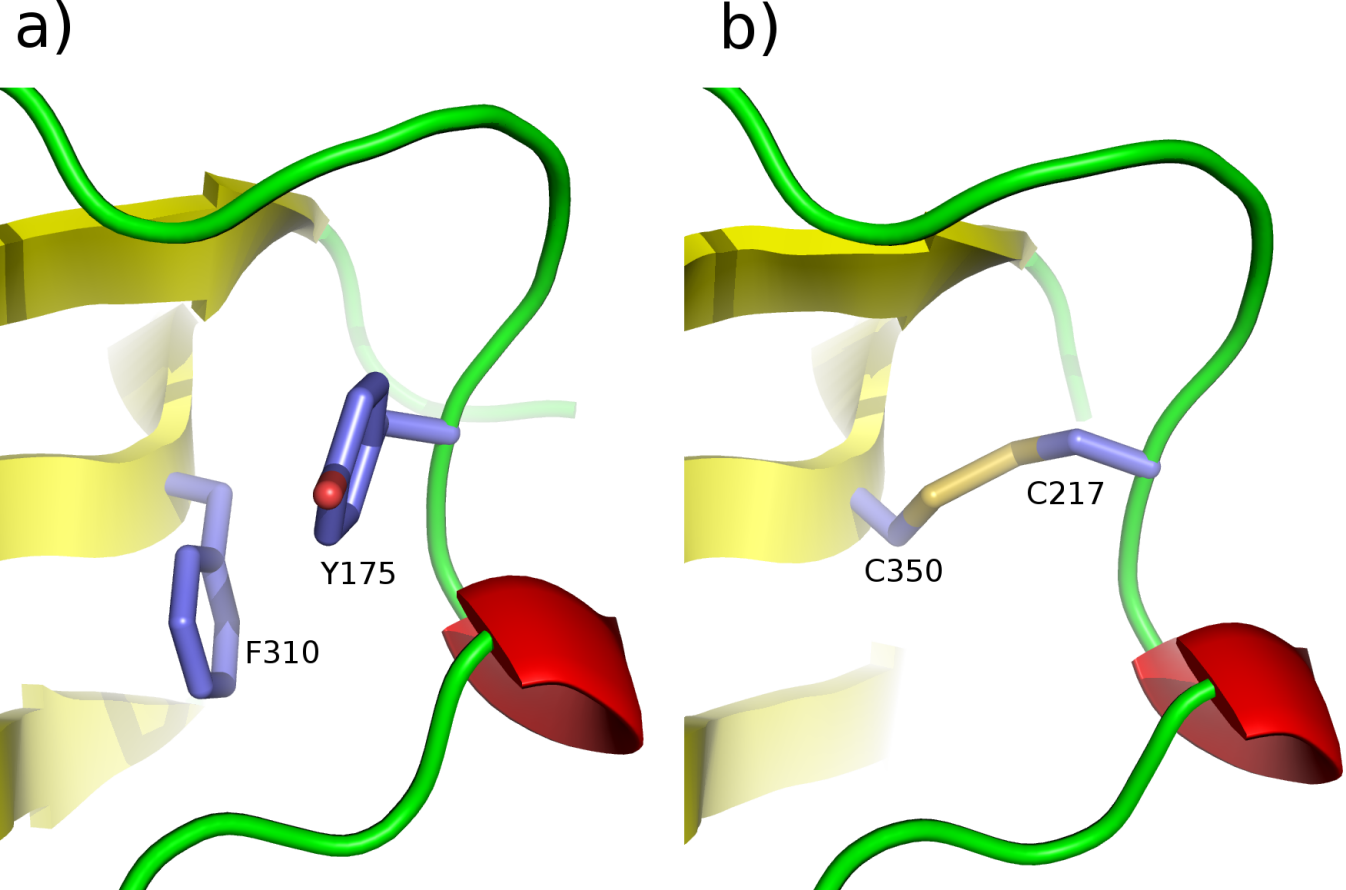
**A putative novel disulphide bond in *S. ratti *ASP-2A**. Comparison of human pepsin template (PDB code 1pso; [44]) and ASP-2A model in the vicinity of the ASP-2A-specific novel putative disulphide bond. The figure was produced using PyMOL [49], as were Figures 8 and 9.

A model of the ASP-2A structure lacking the Cys-rich insertion was readily obtained, no doubt helped by the small size of the insertions and deletions in ASP-2A compared to the templates. Using a multi-template approach three insertions of 1, 3 and 4 residues were modelled along with four small deletions of 1 or 2 residues (Figure 5). The model was stereochemically sound with no residues in Ramachandran-disallowed areas and 91% of residues in core regions of the Ramachandran plot. It had a DOPE score, an effective measure of protein structure quality in terms of residue contacts and solvent exposure, of -36610. This compares favourably with DOPE scores of -40670 and -39370 for the similarly sized main templates. Support for the accuracy of the alignment used comes from the observation that Cys residues 217 and 350, neither of which aligned with Cys residues in the templates, were found to be ideally positioned to form a novel disulphide bond (Figure 6).

The 30 residue Cys-rich insertion is far beyond the size limit of around 10-12 residues up to which reasonably accurate loop modelling is considered possible. Nevertheless, modelling was attempted using the predicted secondary structure (20 residues were predicted to adopt α-helical conformation; Figure 5) and predicted disulphide bonding pattern as additional constraints. Unfortunately, there was no agreement between the results of the three disulphide bond predictors: each top prediction contained a different set of three predicted disulphide bonds. However, since the first three Cys residues of the insertion were predicted to lie within a single helix followed immediately by the fourth Cys residue, certain disulphides were rendered sterically impossible if the secondary structure prediction was assumed accurate. Models were therefore made using the insertion disulphide connectivities of (1-2), (3-4), (5-6), the top prediction of DIANNA [17] and (1-2), (3-6), (4-5), the top scoring result from the Cysteine Separation Profile search algorithm at the PreCys server [18]. However, despite extensive efforts no model without obvious defects such as knots could be obtained so further analyses were applied to the model lacking this portion.

**Figure 7:**
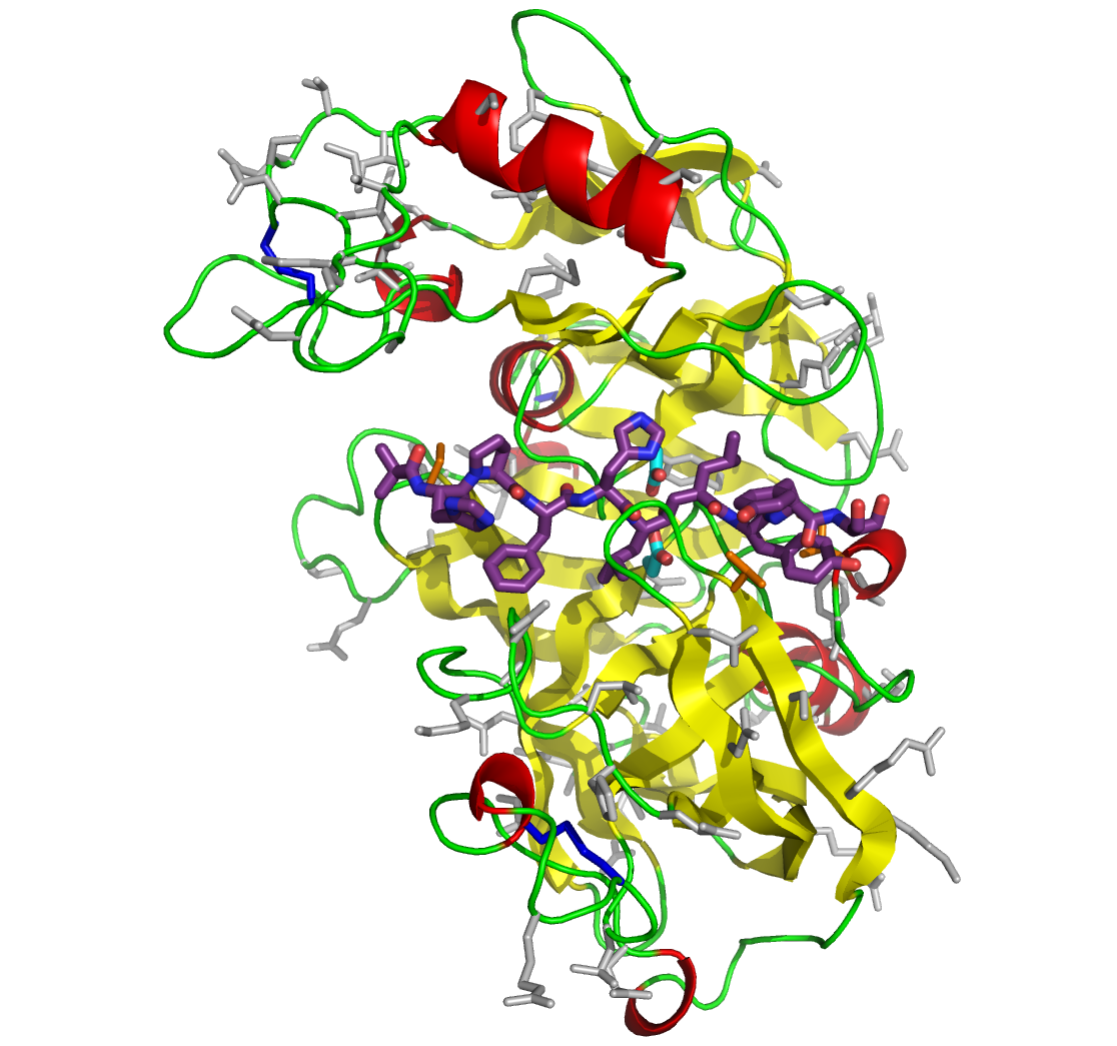
**Cartoon view of the final ASP-2A model coloured according to secondary structure (yellow β-strand, red α-helix and green irregular)**. For the purposes of orientation, the inhibitor from superimposed mouse renin template is shown as purple sticks. Catalytic aspartic acid residues are shown as cyan and red sticks and disulphide bonds as dark blue sticks. Side chains that differ in ASP_2B are shown as orange (near catalytic site) or grey (elsewhere) sticks.

Of the 335 modelled residues of ASP-2A, 74 differ in ASP-2B. Mapping of these on to the ASP-2 modelled structure (Figure 7) shows that they contain more solvent-exposed residues than buried ones so that the 3D structures of the two isoforms may be assumed to be very similar. The differing residues are well dispersed over the molecular surface but, importantly, only three positions are predicted to lie near to the substrate-binding cleft. These are Met15, Thr79 and Ile136 in 2A and are replaced by Ile, Gln and Leu respectively in ASP-2B. These are small to moderate differences in size and physicochemical characteristics, suggesting that substrate specificity will be very similar between the proteins

**Figure 8:**
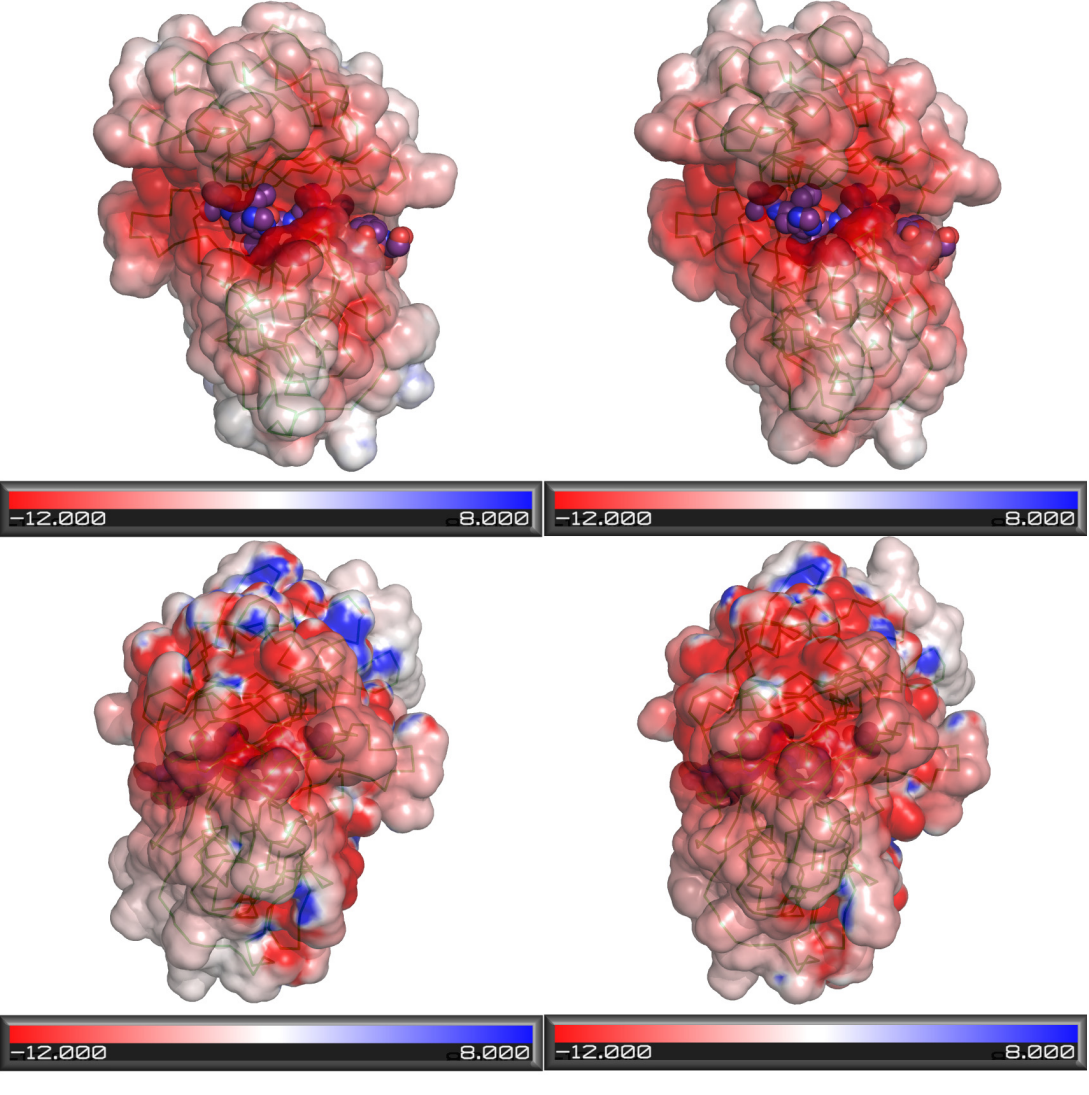
**Comparison of electrostatic surfaces of models of ASP-2A (left) and ASP-2B (right)**. Upper panels show the side of the molecule containing the catalytic site: the inhibitor from superimposed mouse renin template is shown in ball and stick representation. Lower panels show the opposite face of the molecules. Red colouring indicates negative charge and blue positive charge. Electrostatic calculations were done with APBS [55].

While analysing the differences between ASP-2A and ASP-2B, it was observed that among the 22 replacements resulting in charge differences there was a marked preference for positive residues in ASP-2A to be replaced by neutral ones in ASP-2B, or neutral positions in ASP-2A to match negatively-charged residues in ASP-2B. At these 22 positions ASP-2A contains 11 positively charged residues and six negatively charged ones, compared to the figures for ASP-2B of two and three, respectively. This results in a net charge difference on the protein as a whole of -6 in favour of ASP-2B. Electrostatic surface mapping of the ASP-2A model shows that it, in common with other aspartic proteases including both templates (not shown) is strongly negatively charged, on both faces of the molecule but in particular at the substrate binding cleft. Comparison with ASP-2B (Figure 8) shows that the latter's additional negative charge is spread over several parts of the molecular surface. Although we were unable to model the Cys-rich insertion, sequence analysis shows that it follows a similar trend towards more negative charge on ASP-2B vs ASP-2A: of the three isoform sequence differences resulting in charge changes, two are from neutral (ASP-2A) to negative (ASP-2B) and the third from positive to neutral.

## Discussion

We found eight distinct asparatic protease genes transcribed by *S. ratti*. A key question is whether such diversity within the asparatic protease family has arisen recently, particularly within lineages leading to parasitic nematodes, or whether this diversity predated the evolution of the nematode phylum and has been preserved since. While based on a possibly incomplete sampling of *S. ratti *aspartic proteases and a possibly incomplete annotation of aspartic proteases in genome sequences, our phylogenetic analysis suggests three clusters of asparatic proteases. Two of these (Clusters I and II in Figure 3) represent anciently derived aspartic proteases and includes ASP-1, ASP-3 and ASP-4 of *C. elegans*, corresponding orthologs for all three of these genes from *S. ratti *and additional orthologous genes in *C. briggsae, Brugia malayi, Onchocerca volvulus, Necator americanus, Ancylostoma caninum, Meloidogyne incognita *and *S. stercoralis*. The remaining five *S. ratti *transcripts form a monophyletic group within Cluster III (at least within those sequences available to the phylogenic analysis). The diversity among these five *S. ratti *transcripts has therefore arisen from a more recent set of duplication events than those present in Clusters I and II. Similarly within Cluster III, independent duplication events in *C. elegans *and *C. briggsae *can be inferred, while aspartic proteases from the strongylids *N. americanus *and *H. contortus *also group together. Thus, the evolution of aspartic proteases in Cluster III appears more dynamic than in Clusters I and II, involving the repeated duplication of asparatic proteases in different lineages.

Two of the *S. ratti *transcripts within Cluster III, ASP-2A and ASP-2B, appear to have arisen from a relatively recent duplication, share 76% amino acid similarity to each other and exhibit a markedly different expression pattern (Figure 4). Thus, ASP-2A is predominantly found in parasitic females and ASP-2B in free-living females, suggesting that these paralogs may have different functions in parasitic versus free-living females. We investigated the structure-function relationship of *S. ratti *ASP-2A and ASP-2B by homology modelling. Since only three of the ASP-2A/ASP-2B sequence differences were located at the substrate binding cleft, and those were relatively conservative changes, it can be concluded that the substrate specificity of the isoforms is likely to be very similar. One possible explanation for the fixation of two copies of the gene with similar specificity may lie with the analysis of their electrostatic properties. Although both are strongly negatively charged at neutral pH, sequence differences consistently led to extra negative change on isoform ASP-2B. It is possible that these electrostatic differences reflect differences in the pH at which the two isoforms operate. Most intestinal proteases from *C. elegans *exhibit optimal activity at pH 5, suggesting that the *C. elegan*s intestinal lumen is mildly acidic [19], which is consistent with the negatively charged surface of *S. ratti *ASP-2A and ASP-2B. Little is known about nematode digestion [20], but it is possible that the pH of the intestinal lumen of *S. ratti *parasitic adults is less acidic than that of free-living adults, perhaps due to different requirements between digestion of microbiota by free-living stages versus host-derived nutrients by parasitic stages. Accordingly, to maintain solubility and activity, the free living-specific isoform ASP-2B, might have adjusted its pI downwards. It also remains possible that the two isoforms differ in their interaction with as-yet unidentified elements of their different environments, since inter-isoform differences are found throughout the surface of the protein (Figure 7).

Notably, *S. ratti *ASP-2A and ASP-2B share a cysteine rich insertion with each other and with ASP-2 of *C. elegans *(Q86NE0), *C. briggsae *(A8XV46), Q9N9H4 of *N. americanus *and Q70JE2 and Q25037 of *H. contortus*. These aspartic proteases from *N. americanus *and *H. contortus *have previously been implicated in parasitism, particularly the digestion of haemoglobin [4,14,21]. Moreover, Q25037 of *H. contortus *is almost exclusively expressed by the blood-feeding stages (late L4 and adults) of *Haemonchus contortus*, and is localised in the lumen of the gut of the adult parasite [14]. Since *S. ratti *does not feed on blood, the role of ASP-2A in *S. ratti *parasitic females remains unclear, but may involve the digestion of other host macromolecules. The Cys-rich insertion, the similarity of which between enzymes illustrates a single evolutionary origin (Figure 2b), shows a rather sporadic distribution in proteases from Cluster III and is also present in the group of Q18020, A8X733 and A8XQ82 which do not reliably fall within any of the three main clusters. Importantly, different phylogenetic methods, including calculation of differences between sequences by both pair-wise and complete deletion methods, yield the same topology. The absence of the expected close relationship between proteases sharing this prominent sequence feature indicates that the insertion must have been present in the common ancestor of them all, but lost several times during evolution. The repeated loss of this prominent feature is itself concomitant with the repeated duplication of genes within Cluster III. This impression of ongoing genetic flux in the nematodes is in accord with previous analyses of other phenomena such as gene duplication and diversification [22], gene movement between chromosomes [23] and gene deletions and truncations [24].

Our results from *S. ratti *demonstrate that transcriptomic and genomic sequence data can be combined to identify distinct members of a gene family. Such data are increasingly becoming available for other parasitic nematodes, particularly in light of concerted efforts by major sequencing centres and with the advent of new pyrosequencing techniques [25]. There will therefore soon be the opportunity to perform comparative evolutionary analyses and to examine the processes of gene gain and loss acting on large numbers of gene families across multiple nematode species. Doing so will provide fresh insights into the genetic and evolutionary mechanisms associated with parasitism. However, such comparative evolutionary analyses will also bring considerable challenges. Although we have used a number of bioinformatic methods to identify and align aspartic protease sequences, these methods have required manual supervision to construct reliable phylogenies. A more automated approach, suitable for analysis of multiple gene families, will still require validation by a manually supervised approach. Our results also highlight the inherent difficulties in imposing a consistent nomenclature on duplicated members of gene families. Ideally, one would wish the nomenclature to reflect the evolutionary relationships between the orthologs, perhaps by associating parasitic nematode genes to their corresponding *C. elegans *ortholog. However, as is the case for *S. ratti *aspartic proteases in Cluster III, gene duplications in lineages leading to parasitic nematodes generate paralogs and so confound a nomenclature based on orthology to a reference sequence genome. Finally, the type of bioinformatic and evolutionary analyses that we present here can highlight genes, such as *S. ratti *ASP-2A, which may have acquired a significant role during the evolution of parasitism. As such, these analyses help guide and inform - but are not a substitute for - more detailed molecular and biochemical work to examine in greater depth the novel functions acquired by such duplicated genes that aid a parasitic life-style.

## Conclusions

*S. ratti *provides a powerful model to explore the genetic adaptations associated with the evolution of nematode parasitism due to the presence of both parasitic and free-living adults within its life-cycle. We have examined the role of gene duplication in adaptation to the parasitic lifestyle by analysis of genes from the aspartic protease family in *Strongyloides *and identified a pair of paralogues differentially expressed in either the parasitic or the free-living phase of the nematode life-cycle, consistent with an important role for aspartic proteases in the evolution of nematode parasitism. While our findings should be tempered by the possible incompleteness of some databases and their annotation, we consider that gene duplication, accompanied by the acquisition of new function, is likely to be more widely applicable to understanding the evolutionary adaptations and biology of nematode parasites.

## Methods

### Collection of *S. ratti *material

A laboratory line (LIV 4) of *S. ratti *was used for all analyses. This line was generated and maintained in female Wistar rats (Charles River, UK) as described previously [26,27]. For the collection of free living stages, faeces from 8-day infected rats was collected and cultured at 19°C [26]. L1, L2 and iL3 stages were removed from the faeces on days 9, 10 and 11 post infection (p.i.) using the methods described by Paterson and Viney [28], whereas free living adult males and females were picked by pipette on day 12 p.i. For the collection of parasitic females, 8-day infected rats were starved overnight and then culled on day 9 p.i. Parasitic females were removed from the small intestines and counted as described by Evans *et al*. [10]. Worms from each stage were placed in 500 μl TRIzol reagent (Invitrogen Life Technologies, Paisley, UK) and stored at -80°C. Total RNA was then extracted using TRIzol Plus extraction columns (Invitrogen Life Technologies, Paisley, UK) following the manufacturer's instructions. All animal work was conducted under United Kingdom Home Office licence (PPL40/2933) and in accordance with the University of Liverpool's ethical review procedures.

### Transcript sequence data from cDNA clones

Transcript sequence data was used from a *S. ratti *cDNA library constructed from 14,761 cDNA clones selected from five stages during parasitic and free-living development as described in Thompson *et al*. [11]. Expressed sequence tags (ESTs), derived by Sanger sequencing, from these clones were assembled into 3,688 unique transcripts or 'contigs', as described by Evans *et al*. [10].

### Transcript sequence data from pyrosequencing

0.7 μg total RNA extracted from adult free living male and female, and parasitic female worms was mixed to form 2.1 μg and then shipped in ethanol to Evrogen Technologies, Moscow. The sample was subjected to SMART cDNA synthesis and amplification [29], followed by Duplex-Specific-Nuclease (DSN) normalisation [30] in order to enrich for full length transcript sequences and equalise transcript abundance. Upon return, 80 ng of normalised sample was subjected to a further round of amplification using the Advantage 2 Polymerase mix and 0.4 μM SMART PCR primer (Clontech Laboratories, France) following the manufacturer's instructions. The resulting product was purified using QIAquick PCR purification columns (Qiagen, Crawley, UK) to a final concentration of ~300 ng/μl prior to pyrosequencing sequencing. At least 4 μg of normalised cDNA was submitted to the Advanced Genomics Facility (AGF) at The University of Liverpool where the sample was prepared using methods essentially as described by Margulies *et al*. [31]. Briefly, sample quality was first assessed using the Agilent bioanalyser and then the sample was nebulised into 300-500 bp fragments, blunted and ligated to short adapters. Each fragment was then fixed to DNA capture beads (one fragment one bead) and subjected to emulsion PCR (emPCR). All fragments were amplified in parallel and then placed into individual wells of a PicoTiterPlate where they were sequenced also in parallel using the Genome Sequencer FLX Instrument (Roche). Contig assembly was carried out using the Roche Newbler assembly programme, software version 1.1.03.24.

### Genome sequence data

Data from an early draft of *S. ratti *genome sequence was used, generated by the Sanger Institute, UK. This consisted of 437,784 shotgun reads comprising 281 Mb of total sequence assembled into 9,792 contigs with a combined length of 37 Mb http://www.sanger.ac.uk/sequencing/Strongyloides.

### Identification of aspartic protease transcripts within *S. ratti *transcript sequence data

Similarity searches were conducted on the consensus sequence of each contig built for the cDNA library and for the results of pyrosequencing to assign putative identities. For this, BLASTx [32] searches with a cut-off of e < 1 × 10^-10^, against wormpep196 http://ftp.sanger.ac.uk/pub/wormbase[33], UniprotKB/Swiss-Prot http://www.uniprot.org[34] and nr http://www.ncbi.nlm.nih.gov[35] were carried out. The databases used were those available during September 2008. Where incomplete protein sequences were obtained, possible erroneous frame shifts were sought using the translate tool at the Expasy website http://www.expasy.org/tools/dna.html and, where found, manually corrected. Only transcripts that could be unambiguously generated in this way were considered for further analysis. In some cases, the 5' end of transcripts were missing and here a BLASTn search with a cut-off of e < 1 × 10^-10 ^was carried out against the draft *S. ratti *genome.

### Identification of aspartic proteases within *S. ratti *genome sequence data

The protein sequence of the ASP-1 for *C. elegans *was used to search against the nr database using a cut-off of e < 1 × 10^-10^. A PSI-BLAST [32] profile was constructed over 4 iterations. This protein profile was then used to search against the *S. ratti *genome draft by PSI-tBLASTn. The gene finder program FGENESH [36] was used to look for possible aspartic proteases in the genome. These approaches were used in order to obtain any genomic sequence which may encode for aspartic protease but not sampled by the cDNA library or by the pyrosequencing method.

### Phylogenetic analyses

The Pfam database http://pfam.sanger.ac.uk/[37] was searched for the eukaryotic aspartic protease domain, PF00026, and all sequences listed under nematode and human branch of the tree were retrieved. Partial sequences were once again removed, as well as redundant sequences and those representing the human cathepsins E (not relevant for the present work). In addition, a rigorous inspection to remove duplicated entries in the database was also performed. However, some entries were retained where it was unclear whether they represented duplicated entries or duplicated genes. Thus, for *C. elegans *and *C. briggsae *aspartic proteases, the 6 well characterised aspartic proteases plus 12 (*C. elegans*) and 11 (*C. briggsae*) putative proteins inferred from cDNA sequences were included in the sequence analysis. We did not include *C. briggsae *entry A8XDC1 which is clearly incomplete. Sequences A8XDB7, A8XDC0 (ASP-5_ Cb) and A8WZH3 (ASP-4_Cb), were included in the phylogenetic analysis although we note there are some doubts regarding the accuracy of these sequences: for example, the sequence of A8XDC0 may contain a frame-shift towards the C-terminus. For *H. contortus*, two entries which has high identity between them (Q25037 and Q9U8G6) were included in the sequence analysis. Amino acid alignments were produced from a total of 68 sequences, and 56 (excluding the human sequences), including eight from *S. ratti *with transcript evidence and 2 inferred by homology in the *S. ratti *genome. Alignments were built using the MUSCLE program [38] and manipulated using the JALVIEW program [39]. Phylogenetic analyses were performed by calculation of a protein distance matrix followed by application of the neighbor-joining method using the MEGA package [40,41]: gapped regions were excluded in a complete fashion and percentage bootstrap values from 500 replicates were derived.

### 5' sequencing by Rapid Amplification of cDNA ends (RACE-PCR)

Using the information given in the contig sequences, gene specific primers were designed to amplify the 5' ends of *asp-2A *and *asp-2B *by RACE-PCR (Additional File 1). 1^st ^strand cDNA was synthesised as described for pyrosequencing. For second strand synthesis, 0.2 μM SMART PCR primer (Clontech Laboratories, France) was used with 0.4 μM gene specific primer to amplify a 200 bp product for both genes. Each product was purified using QIAquick PCR purification columns (Qiagen, Crawley, UK) and 100 ng sent to MWG-Biotech, Germany for sequencing.

### Analysis of transcription abundance by realtime PCR (rtPCR) analysis

1^st ^strand cDNA was produced using total RNA from the different *S. ratti *life stages following protocols previously described [10]. Oligonucleotide primer pairs specific for each aspartic protease gene were designed (Additional File 1) using the Primer Express™ program (Applied Biosystems, Warrington, UK) to amplify a 60-90 bp insert with an annealing temperature of 60°C. Primers specific for the small ribosomal subunit S20 (rpl20) gene were also designed and used as a reference for normalisation of the data [10]. rtPCR analysis was performed on all samples using protocols also previously described [10] and for each life stage the relative abundance of each transcript was calculated. Three technical replicates were performed for each gene on all samples. Biological replication was available for the L1, L2, parasitic females, free living females and free living males (two replicates each) but not for the iL3s.

### Molecular Modelling

The profile-profile matching server HHPRED [42] was used to select the most favourable templates for model building of ASP-2A from the many available aspartic protease structures. It showed that pepsins were the most closely related available structures followed by renins. Complexed structures, of human pepsin in complex with pepstatin (Protein Data Bank [PDB; [43]] code 1pso; [44]) and of mouse renin bound to the decapeptide inhibitor CH-66 (PDB code 1smr; [45]) were chosen as the principal templates. The pepsin was 38% sequence identical to ASP-2A, while for renin the figure was 34%. For the bulk of the ASP-2A model structure both templates were simultaneously used, but they differed in the size of several loop regions. In those places a single template was used based on local sequence similarity with ASP-2A. For another loop a lower-ranked structure, *Candida albicans *aspartic proteinase 3 (PDB code 2h6t; [46]) was used to provide a loop of the same length as ASP-2A. For a further region the structure of human renin (PDB code 1hrn; [47]) was used to provide a template where a portion of the mouse renin structure had not been localised in electron density maps. The positions of insertions and deletions in ASP-2A with respect to the templates were inspected and, where necessary, suitably manually repositioned. Templates were superimposed using SSM [48] and inspected using PyMOL [49]. Manual adjustments to the HHPRED-derived sequence alignments of ASP-2A with templates were made using JALVIEW [39].

Modelling was carried out with MODELLER 9v5 [50] initially for the ASP-2A sequence lacking the Cys-rich insertion. In the first models it became apparent that Cys217 and Cys350, neither aligning with Cys residues in any of the templates, were suitably positioned to form a disulphide bridge. This bond was manually specified in a second modelling round during which ten models were constructed. These derived from a 3Å coordinate randomisation procedure applied to the initial crude model generated by MODELLER. This is an established method for exploring conformational space. The stereochemical quality of the models was assessed with PROCHECK [51] and the single model without any residues in disallowed regions of the Ramachandran plot was selected. Constraints on the structure of the Cys-rich insertion that could help modelling were sought from two sources - predictions of disulphide bonding patterns (based on the reasonable assumption that the Cys residues in the insertion form intramolecular disulphide bonds in the extracellular environment) and predicted secondary structure. Disulphide bond predictions were obtained using DiANNA [17], DISULFIND [52] and PreCys [18]. A secondary structure prediction from PSIPRED [53] was obtained at the Meta server [54]. These constraints were incorporated into modelling of the complete ASP-2A sequence using MODELLER.

## Authors' contributions

LVM designed and performed the sequence and phylogenetic analysis and drafted the manuscript. HE performed the PCR analysis. DJR did the protein modelling study. SP conceived and planned the project. DJR and SP contributed to the analysis and interpretation of the data. All the authors read and approved the final manuscript.

## Supplementary Material

Additional file 1 **Supplementary Table 1**. Primer sequences used in study. Click here for file
