# Supplementary material for: Identification of novel aspartic proteases from Strongyloides ratti and characterisation of their evolutionary relationships, stage-specific expression and molecular structure
Source: BMC Genomics. 2009 Dec 16;10:611. doi: 10.1186/1471-2164-10-611 (PMC2805697; doi:10.1186/1471-2164-10-611)
Supplement: Additional file 1 — Supplementary Table 1. Primer sequences used in study. [file 1471-2164-10-611-S1.DOCX]

Supplementary Table 1. Primer sequences used in study.

| **Gene** | **Forward Primer** | **Reverse Primer** | **Annealing temperature** |
| --- | --- | --- | --- |
| **Realtime PCR** | | | |
| **Aspartic protease** | | | |
| *asp*-*1* | TGCCACCTCAATTCTGAAG | TGCTGGCCATCCTAATCCA | 60 |
| *asp*-*2A* | TGATGATTTTAAGAATGATCCAGC | ACCATCAACTGAAATCGAAGG | 60 |
| *asp-2B* | TTCGTAGGAAGCAAAGGAAATCT | GCAAAATCAGAAGAAATTTGTTGG | 60 |
| *asp-3* | CAAGTTTTCGCTTTCTGGCTTAA | TCACAAAGTGTCATTTCTCCTCCTT | 60 |
| *asp-4* | AAATGTTACAGCAATGGGTAAGTCAA | CACACGTTCAGGAAGATCCATC | 60 |
| *asp-7* | CAATGATGAATATAAAATTTACACAG | CTCCCTAAATTAACGTCATTGATA | 60 |
| *asp-9* | CATAGAGGATATGGAATGTACACAA | CTCCCACACCAGTATGATCAACA | 60 |
| **Reference gene** | | | |
| *Rps-20* | CACGTAAACTCAGAGGACATGTC | CAAGATGCAAGCTCCACCAG | 60 |
|  |  |  |  |
| ***asp-*2 5' RACE** | | |  |
| *asp-2A* | - | ATCTTTGTATTTCAAATACTCTTGC | 51 |
| *asp-2B* | - | ACTTGTTGTTTTGCTTGGTG | 51 |
| 5' Primer | AAGCAGTGGTATCAACGCAGAGT | - | 51 |
| 3' Adaptor | ATTCTAGAGGCCGAGGCGGCCGACATGTTTTTTTTTTTTTTTTVN | - | - |
| 5' Adaptor | AAGCAGTGGTATCAACGCAGAGTGGCCATTATGGCCGGG | - | - |
|  |  |  |  |
| ***asp-7 and 9* Internal PCR** | | |  |
| *asp-7* | CTAGGAAATATATCAATTGGAACTCC | CTCCCTAAATTAACGTCATTGATA | 60 |
| *asp-9* | CTAGGAAATATATCAATTGGAACTCC | CTCCCACACCAGTATGATCAACA | 60 |
